# Polysaccharides in Ocular Drug Delivery

**DOI:** 10.3390/pharmaceutics12010022

**Published:** 2019-12-24

**Authors:** Natallia Dubashynskaya, Daria Poshina, Sergei Raik, Arto Urtti, Yury A. Skorik

**Affiliations:** 1 Institute of Macromolecular Compounds of the Russian Academy of Sciences, Bolshoy pr. V.O. 31, 199004 St. Petersburg, Russia; dubashinskaya@gmail.com (N.D.); poschin@yandex.ru (D.P.); raiksv@gmail.com (S.R.); 2 Institute of Chemistry, St. Petersburg State University, Universitetskii pr. 26, Petrodvorets, 198504 St. Petersburg, Russia; arto.urtti@helsinki.fi; 3 Division of Pharmaceutical Biosciences, Faculty of Pharmacy, University of Helsinki, P.O. Box 56, FI-00014 Helsinki, Finland; 4 School of Pharmacy, Faculty of Health Sciences, University of Eastern Finland, 70210 Kuopio, Finland

**Keywords:** eye tissue regeneration, intravitreal administration, ocular bioavailability, ocular drug delivery, periocular administration, polysaccharide, topical administration

## Abstract

Polysaccharides, such as cellulose, hyaluronic acid, alginic acid, and chitosan, as well as polysaccharide derivatives, have been successfully used to augment drug delivery in the treatment of ocular pathologies. The properties of polysaccharides can be extensively modified to optimize ocular drug formulations and to obtain biocompatible and biodegradable drugs with improved bioavailability and tailored pharmacological effects. This review discusses the available polysaccharide choices for overcoming the difficulties associated with ocular drug delivery, and it explores the reasons for the dependence between the physicochemical properties of polysaccharide-based drug carriers and their efficiency in different formulations and applications. Polysaccharides will continue to be of great interest to researchers endeavoring to develop ophthalmic drugs with improved effectiveness and safety.

## 1. Introduction

Drug efficacy and safety are essential prerequisites for any pharmaceutical product. However, the desired efficacy and safety conditions are often difficult to achieve in ophthalmological settings due to limitations in drug delivery associated with the route of drug administration and imposed by the barriers related to the anatomy and physiology of the eye. For this reason, polymeric carriers and excipients are now being used to enhance ocular drug delivery, and several reviews have described the use of synthetic and natural polymers for this purpose. For example, Formica et al. [1] reviewed the properties of natural polymers and the use of natural polymers for the preparation of nanocarriers for topical ocular drug administration. Similarly, Nayak et al. [2] discussed the properties of liposomes, emulsions, nano- and microspheres, and conjugates as ophthalmic drug formulations, although the topical and intravitreal systems used in that study were based on non-natural biodegradable polymers, such as poly(lactic-*co*-glycolic acid) (PLGA), polyethylene glycol (PEG), and polyvinyl alcohol. Tsai et al. [3] examined the use of the polysaccharide chitosan in eye drops for DNA delivery to the eye, but no reviews have yet explored the effectiveness of drug delivery to various eye segments in terms of the properties of polysaccharides and their derivatives, nor have ophthalmic drug delivery systems based on polysaccharides been described in detail. The aim of this review was therefore to summarize the current advances being made in the development of ocular drug carriers based on natural and semi-synthetic polysaccharides, with emphasis on the properties of polysaccharides as drug carriers and their biological interactions in the eye. 

**Figure 1:**
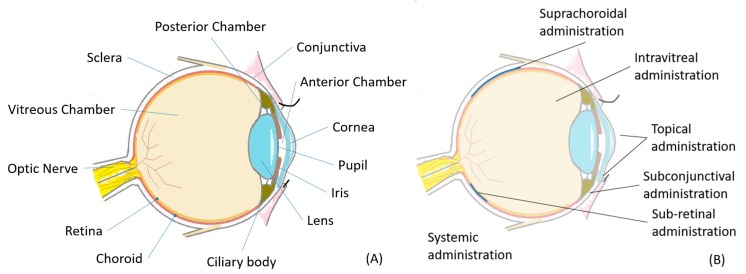
Schematic representation of the anatomical structure of the eye (**A**). Schematic representation of drug delivery routes to the eye (**B**).

The human eye has a complex anatomical and physiological structure that includes both mechanical and chemical barriers that complicate drug delivery to the eye (Figure 1A). Three main routes are available for local ocular drug delivery: topical, periocular (subconjunctival, sub-tenon, peribulbar etc.), and intravitreal (Figure 1B). Some rarely used additional routes of local ocular drug delivery (including subretinal and suprachoroidal) are also available (Figure 1B). The drug bioavailability depends on the route of drug administration into the eye. Therefore, the anatomical and physiological features of the eye must be taken into account when designing drug delivery systems. 

Ocular drugs can be administered systemically (as parenteral injections or by the oral route). The main advantages of systemic over intraocular drug delivery include the convenience of drug administration and greater ocular safety; however, the required drug doses, and therefore the risks of systemic adverse effects, are increased [4]. The delivery of systemically administered drugs to the retina is limited by various factors, including gastrointestinal barriers for the oral route, wide distribution of the drug to off-target sites, and the presence of the blood-retinal barrier [5]. These limitations mean that systemically administered drugs have extremely low ocular bioavailability and must therefore be administered as large daily drug doses [6,7]. This review will focus on the use of polysaccharides as vehicles that can facilitate locally administered drug delivery. 

## 2. Polysaccharides Used in Ophthalmology 

Natural polysaccharides are attractive options for the formulation of ocular medications, as these polysaccharides are economical, readily available, non-toxic, potentially biodegradable, generally biocompatible, and typically amenable to chemical modifications [8,9,10,11,12,13]. Indeed, chemical modifications have recently led to derivatives with improved properties in terms of rheology, mucoadhesion, increased ocular retention, and drug solubilization [14,15,16,17,18].

Currently, the Food and Drug Administration (FDA) allows clinical ophthalmic use of the polysaccharides listed in Table 1. Most of these are presently added to commercially available eye drops as inert thickening agents, although some are used for eye surgery (e.g., hyaluronic acid) or as intravitreal implants (e.g., microcrystalline cellulose). In the coming years, this list will probably expand [19,20].

Cellulose derivatives were the first group of polymers to be used as components in topical ophthalmic dosage forms, and cellulose-based materials remain the most widely used polymers in ophthalmology [21]. Pure cellulose is not water soluble due to its relatively high crystallinity, so cellulosic derivatives were first used as viscolizers in eye drops. These derivatives included methylcellulose, hydroxyethyl cellulose, hydroxypropyl cellulose, hydroxypropyl methylcellulose (HPMC), and carboxymethyl cellulose (CMC). The main disadvantage of these derivatives is that defining a boundary between viscous solutions and an undesired gel phase is challenging because data on polymer concentrations or the formulation viscosities are not always available. Nevertheless, many cellulosic polymers are currently components of ocular products (Table 1). 

The types of polysaccharides used in these products have recently been extended to include other compounds, such as chitosan and its derivatives, hyaluronic acid (HA), and alginic acid (ALG). These are sometimes preferred over cellulosic derivatives for certain applications. 

HA is a natural polysaccharide found in the skin, connective tissues, umbilical cord, and the vitreous body of the eye. The main advantages of HA include its excellent biocompatibility and mucoadhesiveness, as well as its pseudoplastic and viscoelastic behavior. Its use as a vehicle in ocular drug delivery has been reviewed previously [23,24]. 

HA is used as the vehicle in tear substitutes, since it has protective effects against damage caused by benzalkonium chloride, a commonly used preservative in eye drops [25]. The extended residence of HA on the ocular surface is one of its advantages for use as an artificial tear component in dry eye therapy. Pseudoplastic behavior is another advantage provided by HA eye drops, as this property ensures good spreading of the polymer on the ocular surface upon blinking. HA can also be cross-linked and formulated as films, inserts, or particles to prolong drug release, precorneal residence, and/or drug action (e.g., the ocular hypotensive effects of timolol) [26]. Some commercial HA-containing products are also available as surgical aids for anterior segment procedures (e.g., cataract extraction and intraocular lens implantation). 

Chitosan is another emerging polymer being considered for ophthalmic use [27,28]. Mucoadhesive chitosan formulations have been investigated as a strategy to overcome the rapid elimination of instilled ophthalmic solutions. Overall, chitosan-based formulations appear less viscous than those based on traditional viscolizers like HA. Another possible advantage of chitosan is the presence of positive charges on its backbone at physiological pH. These positive charges are thought to interact with the negative charges of the ocular mucus, thereby enhancing bioadhesion. Chitosan formulations have shown promising results due to their significantly longer retention on the corneal surface when compared with conventional commercial ophthalmic solutions [27,29,30].

ALG is a seaweed-derived polysaccharide composed of β-d-mannuronate (M) and α-l-guluronate residues (G). Solutions of ALG can be crosslinked to form a hydrogel by a mild gelling reaction that involves exposure to divalent or trivalent cations [31]. Most commonly, gelation is achieved using Ca^2+^ ions, usually by immersion of the ALG in a bath of CaCl_2_ to elicit the mild gelling reaction. The Ca^2+^-induced crosslinks are known to dissipate from the scaffold over time in response to exposure of the scaffold to other ions (e.g., Na^+^, K^+^, Mg^2+^, or phosphates), and this results in scaffold degradation [32]. The mechanical properties of ALG gels can be tailored by altering various features, including the molecular weight (MW) of the polymer, the G:M ratio, the cross-linking species, and the concentration of cross-linking cations [33,34]. 

Pectin is a natural polymer constituent of many plant cell walls, and it is extensively employed in the food industry and biomedicine. It is a branched macromolecule with a high MW, so pectin can be converted into swellable hydrogels, particularly from solutions with high pectin concentrations and at low pH conditions, as these conditions facilitate the formation of the coil entanglements responsible for gel formation. Water-insoluble gels can also be obtained by cross-linking with divalent or trivalent cations. Depending on the degree of esterification, pectins are classified as low methoxyl (<50%) or high methoxyl (>50%) forms that have dissimilar gelling properties [35]. The use of pectin hydrogels has been evaluated as a strategy for enhancing drug contact time and ocular absorption. An alternative approach is to apply pectin for in-situ gel formation. In the eye, pectin applied in a liquid form will spontaneously form a gel in response to the neutral lacrimal pH and the presence of the lacrimal electrolytes [36]. 

Other polysaccharides have also been evaluated as potential vehicles for prolonging the residence time of drugs at the surface of the eye. Xanthan gum is one example of a negatively charged polymer that has been proposed as a material for artificial tear preparations [37], and as a vehicle for drug delivery [38]. For example, Saettone et al. [39] evaluated the transcorneal delivery of pilocarpine from several ophthalmic formulations and demonstrated that 1.5% xanthan gum significantly improved both pilocarpine bioavailability and the duration of the drug action. 

## 3. Topical Administration

### 3.1. Barriers to Topical Delivery of Ocular Drugs

Topical instillation is the most common ocular drug delivery method, particularly for outpatient treatments. However, drug bioavailability to the anterior eye segment (cornea, aqueous humor, iris, and ciliary body) is generally less than 5%, and it is 2–3 orders of magnitude lower still in the posterior segment, which contains the retina. The inability to deliver drugs by topical and systemic routes and/or the inefficiency of those routes for retinal delivery of existing drugs is now widely accepted [40]. The low levels of drug absorption are due to the presence of anatomical and physiological barriers that include nasolacrimal drainage, the corneal barrier, and non-target absorption of drugs by the conjunctiva [41]. Ocular absorption can be increased by prolonging the residence time of the formulation on the ocular surface using ointments or polymeric inserts but increased ocular contact time with ointments has led to blurred vision and polymeric inserts are often ineffective due to a lack of patient compliance. Thus, new systems are needed that will provide prolonged drug action and better patient compliance. 

Drug bioavailability from eye drops is hampered by ocular barriers that limit drug exposure to the ocular surface and prevent permeation into the target region of the eye [42]. The cornea is the main route of drug absorption into the ocular tissues [43], but the permeability of the cornea to a drug instilled as an eye drop depends on the drug properties, such as its lipophilicity, hydrogen bonding capability, and molecular size [44,45]. The physical features of the formulation itself can also affect drug availability. For example, exposure of the eye to a hypotonic ophthalmic solution causes an increase in the permeability of the corneal epithelium, with a possible triggering of edema. Conversely, exposure to a hypertonic solution has a dehydrating effect. However, in practice, this response is not necessarily strict, as many eye drops are hypertonic and the eye can withstand hypotonic solutions [46].

Control of the formulation pH is also important for drug absorption, since corneal permeation is higher for non-ionized forms of drugs than for ionized forms [47]. In addition, pH is often important for stability of the drug in the formulation. Many formulations have an acidic pH range (3.5–6.3), so they can cause an initial, but usually acceptable, discomfort upon application (the normal lacrimal pH is 7.4) [48]. Similarly, the administration of solutions that have a surface tension lower than that of the tears may destabilize the lacrimal lipid film and disperse it as lipid droplets. The integrity of the oily film formed by the lacrimal fluid is essential because it reduces the rate of evaporation of the water layer, thereby improving the retention of water in the tear film and preventing dry eye [49]. 

### 3.2. Eye Drops and Artificial Tears

Viscosifying agents are used in ophthalmic solutions to prolong the retention time of the drug on the surface of the eye. In general, a higher viscosity leads to higher precorneal retention times. On occasion, viscous solutions are poorly tolerated by patients because of shearing forces that occur during eye movements and blinking. Therefore, pseudoplastic (shear-thinning) properties are advantageous in formulations intended for topical administration [50]. In addition to increased viscosity, factors like mucoadhesion and comfort upon instillation play further significant roles [51]. Several polymers, including chitosan, HA, ALG, cellulose derivatives, arabinogalactan, xyloglucan, gellan gum, and their mixtures, have been proposed for eye drops and for in-situ gelling systems. Among these polysaccharides, chitosan shows the best mucoadhesion because of its cationic nature. 

Different polysaccharides, such as ALG, tamarind seed polysaccharides (TSP), HA, and hydroxyethyl cellulose, have been compared for their ability to resist removal from tear fluid. Their mucoadhesivity has been examined both in vitro (using the polymer-induced increase in the viscosity of a mucin dispersion) and in vivo (using the polymer residence time in rabbit tear fluid) [52]. An optimal excipient polymer in eye drops should be mucoadhesive but not excessively viscous when in solution. A solution of TSP (0.7% *w*/*v*) had the highest mean precorneal retention time in the rabbit eye. By virtue of its mucoadhesivity, TSP (0.7% *w*/*v*) also prolonged the residence of the drugs ketotifen fumarate and diclofenac sodium in the precorneal area of rabbit eyes [52]. HA and hydroxyethyl cellulose are also mucoadhesive, but their solutions are too highly viscous and could cause discomfort to the patients by potentially inducing reflex tearing. At similar concentrations, ALG did not show significant mucoadhesion, but it did not increase the solution viscosity, so it can be used at higher concentrations (up to 5–10%) to provide mucoadhesion [52,53]. 

Polymer solutions are used for their interactions with the ocular surface, which prevent the loss of cellular volume while suppressing cellular stress and inflammatory reactions. For example, a combination of CMC and HA (a natural component of the tear film) improves the water retention and viscoelasticity of the lacrimal film [54]. A double-blinded multicenter study of 305 subjects demonstrated that an artificial tear formulation of a novel multi-ingredient formulation provided the best improvement in ocular symptoms and ocular staining. This formulation contains 0.5% CMC, 0.1% HA, salts, osmoprotectants (glycerin and erythritol), and a stabilized oxychloro complex (for preservation) [55]. Treatment with a lubricant eye drop containing PEG, propylene glycol, and hydroxypropyl guar preserved with polyquaternium-1 also reduced the severity of dry eye disease (DED) and surface inflammation by reducing the number of cells with D-related human leukocyte antigen on the ocular surface [56]. D-related human leukocyte antigen plays an important role in T-cell activation and is overexpressed in many patients with DED [57]. Assessments conducted in that study included DED severity, corneal and conjunctival staining, tear film breakup time, Schirmer tests, and impression cytology of the conjunctiva with masked flow cytometry at baseline and 30 days. 

Another tested artificial tear preparation contained two active ingredients: HA and trehalose [54]. In general, trehalose stabilizes lipid membranes and proteins against desiccation, and it does so in the eye as well. These artificial tears were compared with a preparation containing hyperbranched polyglycerol and polyquaternium-1 preservative, and both formulations were found effective for the treatment of DED in blinded multicenter studies with patients with dry eye symptoms. The effects on tear breakup time and ocular surface damage have also been measured after delivery of formulations containing CMC, glycerol, polysorbate, and emulsified lipid [55]. The lipid-containing artificial tears could also be used to counteract the lipid deficiency associated with DED. 

One study focused on maximizing the viscosity and mucoadhesiveness of HA-based preparations [58] by examining the range of MWs encountered in commercial products. The concentrations varied from 0.3 wt% for an 1100 kDa HA, up to 1.0 wt% for a 250 kDa HA (i.e., three-fold higher than the highest concentration in the clinical products). The viscosity and mucoadhesion profiles of the optimized formulations were superior to those of the commercial products, especially under conditions that simulated in vivo blinking, and therefore predicted longer retention on the corneal epithelium. An enhanced capacity to protect corneal porcine epithelial cells from dehydration was also demonstrated in vitro. Overall, the results predicted formulations with improved efficacy. 

A dual-polymer formulation consisting of hydroxypropyl guar and HA was as effective as HA lubricant eye drops for improving ocular surface staining in DED. The hydroxypropyl guar-HA formulation did not improve the scores for everyday life treatment satisfaction in patients with DED when compared to HA, but no new safety concerns were reported [59]. 

Excipients have also been used in eye drops to increase ocular bioavailability. These materials include micelles [60], nanoparticles, mucoadhesives, in-situ-forming hydrogels, and contact lenses [61]. Note that the use of natural polymers, such as ALG, Veegum^®^, xanthan gum, gelatin, acacia gum, and tragacanth gum, may carry a high risk of bacterial and fungal contamination [62].

### 3.3. Mucoadhesives

Mucoadhesion refers to the adhesion of an exogenous material to the mucin on an epithelial surface, for example, in the eye. Mucoadhesion is explained by four main mechanisms: electrostatic, adsorption, wetting, and diffusion [63]. In general, mucoadhesion is a combination of many processes. First, the polymer swells, as described in the wetting theory, and then non-covalent (physical) bonds are formed at the mucus-polymer interface (electrostatics and adsorption). Thereafter, the chains of the polymer and mucin interpenetrate and entangle to form more bonds via the four mechanisms [63,64].

Many polysaccharides are mucoadhesive at comparatively low viscosities, making them suitable for prolongation of eye drop contact time and for the treatment of DED. Mucoadhesion allows the drops to stabilize the tear film and prolong the residence time of ophthalmic drugs on the eye surface. Chitosan and some of its polycationic derivatives display mucoadhesion and promotion of ocular drug absorption via reversible permeabilization of the cornea [27]. This process has been utilized in further development of chitosan as component of nanoparticles and in-situ gelling systems [65]. In contrast, Rodriguez et al. [66] have indicated that mucoadhesive polysaccharides (chitosan, HA, and ALG) significantly reduce the permeability of the anti-glaucoma drug timolol maleate through ex vivo bovine corneas. The addition of permeability enhancers (like ethylene glycol-bis(2-aminoethylether)-*N*,*N*,*N*′,*N*′-tetraacetic acid or hydroxypropyl-β-cyclodextrin) led to a further reduction in drug permeability when compared to the polymers alone. These results highlight the need for the careful selection of additives intended for ocular applications, since interactions between them can have opposite results to those expected in terms of drug permeability [66].

The use of polysaccharides, such as ALG or gellan gum, as the basic excipients of in-situ-gelling systems revealed that the basic principles underlying any increase in drug bioavailability were the prolonged retention of the gel in the precorneal area and the slow release of the drug from the gel. Bravo-Osuna et al. [67] used surface plasmon resonance to study the mucoadhesive properties of various polysaccharides toward ocular mucins. Chitosan, being a cationic polymer, was found to form ionic bonds with anionic sialic acid and sulfonic acid groups, whereas the primary forces for retention of anionic polysaccharides, such as CMC and HA, were hydrogen bonding and interpolymer diffusion. 

Eye drop formulations have been prepared using HPMC (0.75%, *w*/*v*) in an isotonic solution incorporating chitosan/sodium tripolyphosphate-HA (chitosan/TPP-HA) nanoparticles loaded with ceftazidime [29]. The viscosity of the nanoparticles and the gels incorporating the nanoparticles were characterized after contact with mucin at different mass ratios to calculate the rheological synergism parameter. Different ratios of the nanoparticle eye formulation to mucin weights gave a minimum viscosity that resulted in a negative rheological synergism. The mucoadhesivity of this novel ocular formulation and its ability to interact with the ocular surface were improved, thereby increasing the drug residence time in the eye. In-vitro release and permeation studies showed a prolonged drug release profile from the chitosan/TPP-HA nanoparticle gel formulation. Furthermore, the gel formulation was not cytotoxic to ARPE-19 and HEK293T cell lines, as evaluated by metabolic and membrane integrity tests. The formulation was stable, and the drug retained its activity, as shown by microbiological studies. Thus, chitosan/TPP-HA nanoparticle eye drop formulations are a promising platform for enhancing the mucoadhesive properties of ocularly delivered drugs. 

Chitosan binds to mucin by electrostatic interactions (i.e., between the positively charged amino groups of chitosan and the negatively charged sialic acid residues of mucin), by the formation of hydrogen bonds, and by hydrophobic forces [64,68]. The mucoadhesive properties of chitosan depend on the MW and viscosity of the polymer, as higher MW chitosan has a greater affinity for mucin. High-mucoadhesion chitosan can contract the mucin gel network to create large pores throughout the gel network. These pores then can promote the diffusion of chitosan-based micro and nanoparticles through the mucus layer [69,70].

### 3.4. In-Situ-Forming Hydrogels

#### 3.4.1. Features of In-Situ-Forming Hydrogels

Ocular in-situ-forming gels are a promising alternative to other drug delivery methods and may overcome the drawbacks of conventional eye drops because they retain the advantages of solutions, such as the ease of administration for outpatients and the prolonged contact time of ointments. However, the use of preformed hydrogels has drawbacks that can limit their use in ophthalmic drug delivery or as tear substitutes. For example, they do not allow accurate and reproducible administration of drug doses, and they often produce blurred vision, crusting of eyelids, and lacrimation following administration. 

In-situ-forming hydrogels are instilled as eye drops, but they immediately undergo gelation upon contact with the lacrimal fluid. These hydrogels are therefore stimulus sensitive, and they differ in this respect from inert hydrogels. They can “sense” changes in environmental properties, such as pH and temperature, and then respond by increasing or decreasing their degree of swelling by adjustments in the intricately entangled polymers strands. These formulations seem particular promising, especially since new “patentable” entities might be obtained through in-depth studies of associations of already well-established products. When supplied as eye drops, an in-situ sensing hydrogel are expected to mix with tear fluid and alter its gelation in response to the physicochemical characteristics of the film, which contains many ions and has an osmotic pressure equivalent to that of 0.9% saline, a temperature of +32 °C, and a pH of 7.4. However, one disadvantage of this approach is that the volume of normal tear fluid is only 7 µL, while eye drop volumes usually range from 30–40 µL. Therefore, the eye drop properties may dominate over the lacrimal fluid, making the impact of lacrimal fluid on gel formation inadequate or too slow.

Several in-situ-forming hydrogels have been tested for topical ocular administration. Notably, chemical modification or graft copolymerization of polysaccharides provides almost unlimited opportunities to combine biodegradability, low toxicity, and mucoadhesivity of different polymers with the functional properties of synthetic polymers. For example, a copolymer of poly(*N*-isopropylacrylamide) (PNIPAAm) with chitosan was used to produce thermosensitive eye drops for topical administration of timolol. The mucoadhesive features of chitosan, along with the gelation of the copolymer at a low critical solution temperature (32 °C) due to the PNIPAAm components, resulted in improved pharmacokinetics compared to commercially available eye drops [71]. The PNIPAAm-HA copolymer demonstrated excellent biocompatibility with retinal pigment epithelial (RPE) cells and elicited only a minor inflammatory response, making this formulation potentially suitable for ocular drug delivery even in the form of intraocular injections [72].

#### 3.4.2. In-Situ-Forming Gels Influenced by Ionic Strength

Ionic-strength-responsive polymers undergo their phase transitions in response to changes in the surrounding salt concentrations (e.g., ionic strength). One example of an ion-responsive material is gellan gum, an anionic polysaccharide produced by the bacterium *Pseudomonas elodea* [73]. After dispersion in aqueous solutions, gellan gum undergoes a liquid-gel transition in response to increases in ionic strength [74]. This sol-gel transition process is induced by the presence of monovalent or divalent ions, such as Na^+^ and Ca^2+^. Other parameters can also influence the phase transition, including the polysaccharide concentration, the temperature, and the nature and concentration of cations. The exceptional rheological properties of gellan gum, such as its thixotropy, pseudoplasticity, and thermoplasticity [75,76], are further advantages that favor its use in ophthalmology. Notably, the fluidity of the solution can be increased simply by shaking or slightly warming the preparation, and the gelation increases in proportion to the concentration of monovalent or divalent cations in the lacrimal fluid. In vitro experiments have demonstrated a greater effectiveness of divalent cations than of monovalent ions in promoting the sol-gel transition. In any case, the in vivo tear conditions (i.e., the concentration of sodium in tears) are sufficient to induce the gelation process. Eye drops containing gellan gum and timolol have received market authorization (Timoptic XE) [77]. 

Carrageenans, a group of water-soluble sulfated galactans extracted from red seaweed, show similar features to gellan gum in terms of their rheology, gelling properties [78,79], and biological safety. This suggests that these polysaccharides could also be interesting polymers that could prolong the residence time of topical ocular formulations [79]. Some authors have suggested that these compounds, because they are strong polyelectrolytes, may have the same underlying gelling mechanism as gellan gum. 

ALG is another anionic polysaccharide that undergoes gelation via interactions with divalent cations and with oppositely charged polymers. Some ALG forms are rich in guluronic acid residues and exhibit a reversible liquid–gel transition after administration. These forms were efficient at reducing intraocular pressure when used as a vehicle to deliver pilocarpine [80,81]. ALG-pectin combinations and thiolated pectins have also been studied. The thiolation of pectin increased gelling behavior, viscosity, and bioadhesive strength, while a combination [82] of pectin and ALG demonstrated good in vitro release characteristics [83]. Microparticles of ALG and chitosan have been prepared and used for the loading of 5-fluorouracil [84]. This microparticle formulation increased the delivery of 5-fluorouracil to the aqueous humor in animal experiments. The enhanced delivery was probably a result of the greater mucoadhesiveness of the chitosan-coated particles as compared to a 5-fluoruracil solution or the uncoated particles. The optimized formulation was non-irritating and well tolerated when tested in rabbit eyes.

#### 3.4.3. In-Situ-Forming Gels Influenced by Temperature

Temperature responsiveness is a useful trigger for in-situ formation of drug delivery gels. In this case, the formulation is in the sol phase at room temperature (20–25 °C), and it solidifies in response to the temperature increase when the polymer is administered to the body (temperature 32–37 °C) [85]. 

Poloxamers are a major example of materials that undergo thermosensitive gelation. These polymers consist of a central hydrophobic segment (polyoxypropylene) surrounded by a hydrophilic part (polyethylene oxide). At concentrations above 20% (*w*/*w*), poloxamers exhibit reverse thermal gelation, but this response can be modulated by the addition of cellulosic derivatives, such as methylcellulose or HPMC, to the formulation [86,87]. The mucomimetic property of poloxamers is supposedly a result of their hydrophobic and hydrophilic sequences, which simulate mucin action by adsorption to the aqueous layer of tears on the hydrophobic epithelium. Since the sol-gel transition takes place as the temperature increases, accidental gelation during conservation may occur. Owing to their protective and mucomimetic action, poloxamers have been evaluated for the treatment of dry eye and as ocular drug delivery systems [88]. 

The semi-interpenetrating hydrogels of polyhydroxyethyl methacrylate and methylcellulose are also thermosensitive. An increase in the methylcellulose content increases the viscous component and provides a better moisture retention capacity of the methylcellulose-containing hydrogels, making them suitable for ophthalmic applications [89]. 

#### 3.4.4. In-Situ-Forming Gels Influenced by pH

All pH-sensitive polymers contain pendant acidic or basic groups that can either accept or release protons in response to changes in environmental pH. This response results in changes in the charge state of the polymer chains, thereby inducing conformational swelling or shrinking. Polymeric hydrogels with weakly acidic groups may swell if the external pH increases, while polymers with weakly basic groups may shrink under the same conditions [90]. The pH-sensitive hydrogels contain polymer chain networks that are crosslinked to each other and surrounded by a salt solution. A change in the solution pH will initiate gel swelling or deswelling. This physical process, in general, is not instantaneous, and modeling the gel swelling/deswelling rate provides a thorough understanding of the gel dynamics. This knowledge is important when hydrogels are used for controlled drug delivery, as the drug may release prematurely during the swelling process. These pH-sensitive systems typically exist as low-viscosity aqueous dispersions, but they undergo spontaneous coagulation in the conjunctival cul-de-sac in response to an increase in the local pH. One example of this is a chitosan/carbopol mixture that is liquid at pH 6.0, but it undergoes gelation in the lacrimal fluid pH of 7.4. This system has been evaluated for the delivery of timolol [91] and ketorolac tromethamine [92]. 

Recall, however, that the volume of tear fluid is only 7 µL and that tear fluid is only weakly buffered. Therefore, the pH change may occur in the tear fluid, rather than in the formulation, after the instillation of a pH-sensitive hydrogel. 

### 3.5. Inserts and Contact Lenses

Ocular inserts were first used in the UK in the early 20th century as local anesthetic eye products. These gelatin-based inserts were placed under the eyelid to release cocaine. Later, in the 1960s, soluble ocular drug inserts were introduced for clinical use in the Soviet Union for the delivery of several ocular drugs, such as pilocarpine. Ocusert was introduced for pilocarpine delivery during 1970s by the ALZA Corporation (Palo Alto, CA, USA). This topically inserted device released pilocarpine for a week. In all these cases, increased ocular absorption and prolonged duration of action was achieved, but these products are no longer used clinically. Difficulties in their clinical use, poor ocular retention, and reduced patient compliance resulted in the withdrawal of these products from the commercial market.

Ophthalmic inserts are defined as preparations with a solid or semisolid consistency, whose size and shape are specially designed for insertion into the eye [77]. These inserts are placed topically in the lower fornix and, less frequently, in the upper fornix or on the cornea. The initial discomfort upon administration is a disadvantage that arises from their solid state; other disadvantages include possible movement around the eye, occasional inadvertent loss during sleep or while rubbing the eyes, interference with vision, and generally difficult placement (and removal, for insoluble devices) [93,94]. Ocusert is a delicate, insoluble, and non-degradable reservoir device that is filled with sufficient pilocarpine for 1 week of use, whereas Lacrisert is a soluble mini rod of nonmedicated hydroxypropyl cellulose that dissolves within 24 h for the treatment of dry-eye syndromes [95]. Ophthalmic inserts are generally classified according to their solubility behavior and their possible biodegradability. In general, prolonged drug duration can be achieved with these types of inserts. 

Soluble inserts will dissolve in the tear fluid after instillation. In this case, lacrimal fluid may penetrate rapidly into the device [96], resulting in fast drug release but potentially blurred vision. Conversely, the glassy constitution of the insert may increase the risk of insert expulsion from the eye. Ophthalmic inserts containing cellulose-based polymers are extensively described in the literature [82]. Ethylcellulose, a hydrophobic polymer, can be incorporated into the formulation to decrease insert deformation and blurred vision [97], but these insoluble inserts must be removed from the eye after drug release. Film forming properties and biocompatibility of cellulose ethers makes them a good excipient for modifying ocular insert properties. The addition of methylcellulose to polyacrylic acid films provided prolonged riboflavin release due to the formation of a hydrogen bond network and a decrease in irritation effects [98]. Abilova et al. [99] prepared films based on chitosan and its blends with poly(2-ethyl-2-oxazoline). These films were mucoadhesive, biocompatible, and capable of providing a sustained drug release when administered topically on the cornea.

Lacrimal fluid contains bicarbonates for buffering the pH to about 7.4, but the capacity of this buffer system is quite limited. Therefore, in vitro release tests from ionizable polymer inserts should be carried out using dilute buffers (e.g., 2 mM) [41], as release tests using regular strength phosphate buffers will result in poor in vivo extrapolation [41]. Insoluble inserts can be classified into two categories: reservoir and matrix systems. Reservoir inserts consist of a central reservoir of drug enclosed in a specially designed semipermeable or microporous membrane, which allows the drug to diffuse from the reservoir at a predetermined zero-order release rate (e.g., Ocusert). Reservoir-controlled release systems may be manufactured in a wide range of geometries, including conventional tablets/pellets, laminated films, and other defined shapes (e.g., hemispheres, cylinders, or rods) using, for example, ALG [100,101,102] and cellulose as polymeric materials [103].

Contact lenses with presoaked drugs have been tested for ocular drug delivery since the 1970s. Soft hydrophilic contact lenses were developed for prolonged release of several drugs, such as pilocarpine, chloramphenicol, tetracycline, and prednisolone sodium phosphate [82]. The polysaccharides used in contact lenses include HA and cellulose derivatives [104]. 

HA is used as a solution for topical artificial tears, and it has proven efficacy in the treatment of dry eye syndrome. Using molecular imprinting strategies, the delayed release of high molecular HA from a daily disposable lens can be achieved at a therapeutic rate of approximately 6 µg/h for 24 h [105]. Similarly, the extended release of 120 kDa HPMC at the rate of 16 μg per day [106] has been reported for the prevention of contact-lens-induced DED. The main drawbacks of using contact lenses as therapeutic materials are their high cost of manufacture and their low drug-loading capacity, which is not sufficient to build up a therapeutic concentration in the eye for most drugs [104]. Contact lenses are also changed frequently, and drug loading should be done professionally, not by the patient at home. Furthermore, drug release from the contact lenses to the storage liquid must be avoided. For these reasons, no drug-containing contact lenses have been launched as clinical products. 

### 3.6. Dispersed Systems

Dispersed systems based on liposomes, nanoparticles, or nanocapsules have been extensively studied for ophthalmic drug delivery, but the development of marketable dispersed products has been challenging. The major issues for this type of delivery system include problems in the percentage of dispersed phase/entrapment coefficient (i.e., how much of the active ingredient can be embedded to the dispersed phase), the stability and shelf life of the product, difficulties in antimicrobial preservation, tolerance of the used surfactants, and, last but not least, the complexity of large-scale manufacture of sterile preparations. A recent publication illustrating the importance of drug loading in topical ocular products showed that the required loading levels are strongly dependent on the drug potency and the desired duration of action [107]. In addition to adequate drug loading, the retention of the administered particles in the conjunctival pouch must also be of sufficient duration to allow drug release from the particles before they are drained away from the precorneal area [82]. Storage of the dispersed system in liquid form imposes additional problems, since the drug will be equilibrated and released to the aqueous storage medium. Freeze-drying is also not an appealing option for topical eye drops, because each eye drop would require reconstitution from the powder form before its instillation into the eye.

**Figure 2:**
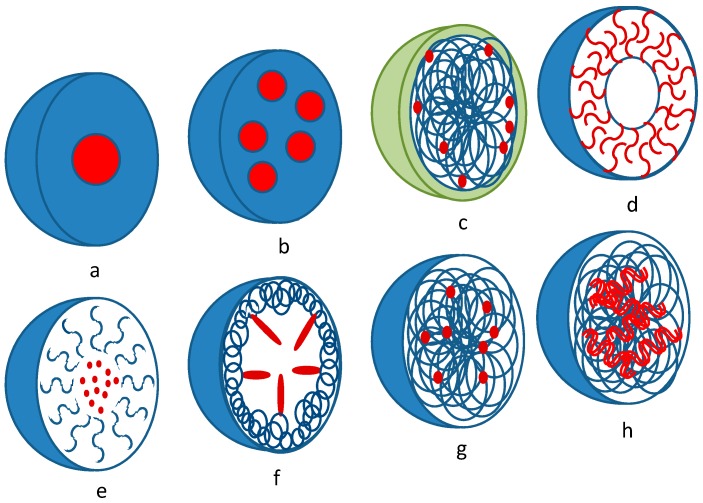
Schematic representation of different types of micro- or nanoparticles: (**a**) core-shell particle; (**b**) multidomain particle; (**c**) layer-by-layer coated particle with encapsulated drug; (**d**) liposome or polymerosome; (**e**) micelle; (**f**) self-assembled particle with conjugated drug; (**g**) micro/nanogel; (**h**) polyplex.

Figure 2 shows the different types of dispersed systems used in ophthalmology for loading with active compounds; their special features are discussed below.

#### 3.6.1. Liposomes

Liposomes (Figure 2d) are microscopic vesicles composed of alternating aqueous compartments and lipid bilayers (mainly phospholipids and cholesterol). The efficacy of liposome use in topical ophthalmic therapy depends on several parameters, including (1) the drug encapsulation efficiency, (2) the size and the surface charge of the vesicles, (3) the distribution of the drug in the liposomes, (4) the stability of the liposomes after instillation, (5) the residence time of the liposomes in the conjunctival sac, and (6) the affinity of the liposomes for the corneal surface [82]. Liposomes and other colloidal drug carriers offer some potential for ocular drug delivery, since they can be used for sustained drug release and prolonged ocular retention. However, liposomes will drain rapidly from the ocular surface if they do not adhere onto the corneal surface. Insufficient residence time of liposomes in the ocular cavity leads to a reduced drug bioavailability, because most liposome-associated drug would be drained away from the eye before its release. Corneal adhesion can be enhanced in different ways, including the use of (1) ganglioside-containing liposomes together with wheat germ agglutinin, a lectin that binds to both the cornea and gangliosides, (2) liposomes coated with antibodies to components in the corneal surface, and (3) liposomes coated with mucoadhesive polymers. 

Polysaccharides have been used to enhance the properties of ocular liposomes. Chitosan enhances the transcorneal permeability of drugs by disrupting the tight junctions in epithelial cellular sheets [27]. Coating of timolol-loaded liposomes with chitosan increased the apparent permeability coefficient of timolol by 2–3-fold when compared to timolol in solution and in uncoated liposomes. The drug retention time in the precorneal area was also increased by the mucoadhesive properties of chitosan [108]. Balguri et al. [109] also reported that ocular delivery of indomethacin was improved with chitosan coated solid lipid nanoparticles. HA has also been used as a coating for lipid-based formulations. This approach increased mucoadhesion of niosomes (non-ionic surfactant-based vesicles) and the ocular bioavailability of the loaded drug tacrolimus [110].

#### 3.6.2. Micro- and Nanoparticles

Micro- or nanoparticles are divided into two groups: micro- or nanospheres and micro- or nanocapsules. Microspheres are monolithic particles consisting of a porous or solid polymer matrix (Figure 2a,b), whereas microcapsules consist of a polymeric membrane surrounding a solid or a liquid drug reservoir (Figure 2c,d) [111]. In practice, the term nanoparticle (or microparticle) is applied both to nanospheres/microspheres and nanocapsules/microcapsules, because the distinction between real capsule structures or matrix-type particles is difficult. The active compound can be dispersed or dissolved in the inner polymer matrix, but it can also be dissolved in the inner reservoir of micelles (Figure 2e), trapped within the particle structure (by gelation, as in Figure 2g, or a layer-by-layer technique, as in Figure 2c), or conjugated to the polymer by forming self-assembling particles (as in the case of hydrophobic drugs). 

Nanocapsules can be used to increase drug absorption from the ocular surface. The aim with these preparations is to increase corneal penetration and prolong the therapeutic response. Corneal penetration of indomethacin was increased 4–5-fold across isolated rabbit corneas when nanocapsules were used as drug carriers [112]. Coating of polycaprolactone-based indomethacin nanocapsules with chitosan, poly(l-lysine), or both, converted the nanocapsules to cationic mucoadhesive formulations [112]. The chitosan-coated nanocapsules enhanced the corneal penetration of indomethacin and showed good ocular tolerance. 

Similar promising findings were also reported for celecoxib-loaded cationic chitosan or anionic ALG nanoparticles, which showed sustained drug release without a burst effect; the release followed a Higuchi non-Fickian diffusion mechanism. These particles were also non-toxic in cell tests [113]. Other reports have investigated the ocular delivery of piroxicam (a non-steroidal anti-inflammatory drug) in pectin microspheres [114]. This system increased ocular drug bioavailability and was well tolerated. 

Yuan et al. [115] prepared self-assembled nanoparticles based on a succinyl-cholesterol derivative of chitosan and used them for topical administration of cyclosporine A. However, isotope label tracking experiments did not reveal significant trans-corneal drug permeation. De Campos et al. [116] encapsulated cyclosporine A in chitosan nanoparticles by ionotropic gelation. In vivo administration resulted in therapeutic drug concentrations in the cornea and conjunctiva. 

Gene therapy is another promising tool for the topical treatment of ocular diseases. Toropainen et al. [117] showed that transfection of corneal epithelial cells can result in the delivery of a secreted transgene product (in this case, secreted alkaline phosphatase) to the anterior chamber. This work demonstrated that topical gene delivery might be used for the treatment of ocular surface layers and anterior chamber tissues. For example, chitosan can be used for DNA binding and condensation. Chitosan’s positive surface charges can prolong the retention of the formulation onto the negatively charged ocular surface. HA can be used to decrease the cationic charge of DNA polyplexes, and it may facilitate CD44-mediated endocytosis into the cells. De la Fuente et al. [118] investigated in vivo plasmid DNA (pDNA) delivery to the corneal and conjunctival epithelia to find that positively charged HA–chitosan/pDNA polyplexes provided efficient transfection by a CD44-mediated mechanism. 

#### 3.6.3. Inclusion Complexes (Clathrates) 

Typically, cyclodextrin-based formulations are used to formulate sparingly soluble drugs, such as carbonic anhydrase inhibitors (for glaucoma) [119] or corticosteroids (for treating inflammation) [120]. However, the action of these formulations might be equivocal in some cases. For example, the use of a cyclodextrin solution of dorzolamide hydrochloride in rabbits resulted in intraocular levels lower than those achieved with the corresponding suspension. On the contrary, promising results were reported for acetazolamide in hydroxypropyl-β-cyclodextrin formulations, as these improved the intraocular pressure [121]. The use of as low a concentration as possible for drug complexation is critical, because the drug must be effectively released from cyclodextrin since these complexes do not permeate into the eye [122]. Too high a cyclodextrin concentration will limit drug release and ocular bioavailability. The best ocular drug absorption can be obtained if a low cyclodextrin concentration (to increase drug solubility) is combined with increased eye drop viscosity (with a polymer solution to increase retention on ocular surface), as shown in vivo with pilocarpine prodrugs [123,124].

In some cases, cyclodextrins enable the formulation of otherwise extremely poorly soluble drugs, such as endogenous cannabinoids (e.g., anandamide) and derivatives that reduce intraocular pressure [125].

Recently, dexamethasone was formulated in cyclodextrin containing eye drop solutions and compared with dexamethasone suspensions [126]. The hydroxypropyl cyclodextrin formulation showed increased dexamethasone solubility by orders of magnitude and resulted in substantial increases in ocular drug absorption, even in the posterior eye segment. 

### 3.7. Summary

Several studies have shown that improvements in topical ocular drug delivery can be achieved with polysaccharide vehicles. Gel forming systems, inclusion complexes of drugs, and nanocomplexes for transfection of gene medicines are particularly promising approaches that are enabling the ocular delivery of difficult compounds. Note, however, that systemic absorption from the ocular surface sets the maximal ocular bioavailability at approximately 10%. Drainage of the formulation and incomplete drug release onto the ocular surface cause further significant reductions in ocular bioavailability, and these factors remain to be optimized. Furthermore, drug release during storage prior to use is a serious limitation in the case of disperse formulations and contact lens-based drug delivery systems.

## 4. Intravitreal Administration

The effective treatment of ocular pathologies requires that therapeutic drug concentrations be achieved and maintained in the target tissues, such as the retina in the posterior segment. Intravitreal injection is an effective means of drug delivery to the posterior segment, but it is an invasive mode of delivery. Currently, more than 22 million intravitreal anti-vascular endothelial growth factor (VEGF) drug injections are given annually worldwide [127]. Even though intravitreal injections are generally safe, the monthly and bimonthly requirements for these injections in long-term treatment may cause problems such as infection and reduced patient compliance [128,129,130,131,132]. For these reasons, intravitreal drug delivery systems are needed that will maintain therapeutic drug concentrations for an extended time, prolong the drug effect, and reduce the required number of applications [128,130,131,133,134,135]. Obviously, the systems must also be safe and ideally they must be biodegradable, as biodegradable drug delivery systems do not require subsequent surgery to remove them from the eye [133].

One of the strategies for long-acting intravitreal drug delivery is to use polymers for extended drug release [130,136]. Intravitreal drug administration requires control of system parameters, such as the size, surface charge, lipophilicity, and shape of drug carriers [137,138,139,140,141], as these parameters affect drug release and retention of the delivery system within the vitreal cavity. The retention of the delivery system and its components within the vitreal cavity depend, in turn, on the physical barriers between the eye and blood circulation and the chemical composition of the vitreous humor [133,141,142,143,144,145,146,147]. The main factors defining the elimination of drug delivery materials from the vitreous humour include: (1) the vitreous humor, (2) the inner limiting membrane (ILM), (3) the blood-retina barrier (including the RPE and blood vessel walls in the retina and ciliary body), and (4) the degradation rate of polymer materials in the vitreous humor [147]. Polymers in the vitreous can be eliminated via the anterior pathway by diffusion from the vitreous into the aqueous humor and then outflow from the eye [148]. Alternatively, the polymers might be eliminated posteriorly, through the vitreous humour, the ILM, and the blood retina barriers, although permeation through this pathway is limited by molecular size [148]. Therefore, particles or polymers are unlikely to escape posteriorly from the vitreous unless they are first degraded into smaller fragments within the vitreous humor [148]. Small molecular weight drugs are mostly eliminated via the posterior route because they are able to permeate through the ILM and blood retina barrier [149].

The vitreous humor has a volume of 4 mL in the human eye [137], and it consists of a negatively charged 3D matrix based on collagen and HA [2,137,143]. The mobility of large particles is hindered due to steric effects, because the limiting mesh size of the vitreous is 550 nm [137,144,150,151]. Large drug molecules diffuse through the vitreous humor slightly slower than small molecules do [2], but the differences in diffusion rate among particles are much greater. The particle surface charge is a more important factor than the particle size in determining the vitreal particle diffusion rate [137,152]. Negatively charged and neutral particles move freely in the vitreous humor, whereas the mobility of positively charged particles is severely limited by the interactions with the anionic components of the vitreous gel [141]. Therefore, hydrophilic, negatively charged or uncharged particles should be used for intravitreal drug delivery [137,147,151,153,154].

HA and collagens are the main components in the vitreous humor, which also contains also a minor quantity of sulfated glycosaminoglycans (chondroitin sulfate and heparan sulfate) [137,143]. The concentration of HA in the vitreous gel is 65–400 µg/mL, and its MW is 2–4 MDa [143]. Vitreous humor collagens (300 µg/mL) are represented by the heterotypic collagen fibrils of Type II, Type V/XI, and Type IX collagens [137,143]. Other structural proteins, such as fibrillin and collagen-binding macromolecules (opticin and VIT1), are also present in small quantities in the vitreous gel [143].

The ILM is an anatomical barrier between the vitreous and the neural retina [141,147,151,155]. It consists of laminin, proteoglycans, and collagen (Type IV), and it limits the access of drug delivery systems and macromolecules to the retina after intravitreal injection [141,147].

The RPE is an epithelial membrane with tight inter-cellular junctions [148] and is located between the neural retina and choroidal blood circulation. The RPE nearly completely blocks the transfer of macromolecules from the retina into the blood circulation [156]. 

### 4.1. Hydrogels

Hydrogels are polymeric depots that physically hold drug molecules to allow controlled drug release [4,136,142,146,157]. Polysaccharides (e.g., HA, ALG, chitosan, and its derivatives) have been used to fabricate hydrogels for intravitreal drug delivery [146]. The hydrogel network is formed by crosslinking macromolecules through chemical (covalent) bonds and/or physical interactions (hydrophobic associations, electrostatic interactions) [146,158]. The resulting hydrogel matrices generate physical or chemical barriers that limit the diffusion of drug molecules, thereby providing the basis for controlled drug release. Drug release mechanisms from hydrogels include diffusion, swelling, degradation, or combinations thereof [146].

Yu et al. [159] used the effective properties of HA (MW 29 kDa) to develop a HA/dextran-based in-situ hydrogel for intravitreal bevacizumab delivery. The HA was chosen as a non-toxic and endogenous ocular polymer. The delivery system was able to maintain a therapeutic concentration of bevacizumab for six months, and the concentration of bevacizumab 6 months after the gel injection was about 10^7^ times higher than the concentration obtained after a bolus intravitreal injection. Clearly, HA based hydrogel formulation has the potential for prolonged dosing intervals of bevacizumab in the treatment of neovascular age-related macular degeneration. 

### 4.2. Dispersed Systems

#### 4.2.1. Micro and Nanoparticles

Dispersed microparticles and nanoparticles have been tested for intravitreal drug delivery. There are two main aims for this approach: (1) prolonged drug retention and release in the vitreal cavity, and (2) improved drug delivery to the retinal and choroidal targets. 

Microparticles improve the drug retention in the vitreous [160]. Sustained drug release from microparticles can be realized by drug diffusion from the polymer or degradation of the microparticles. Accordingly, the time and rate of release can be controlled by the size of the microparticles, the thickness of their walls, and the nature of the polymers [161].

Elsaid et al. [162] studied ranibizumab delivery using a “system-within-system” of PLGA-microspheres that contained nanoparticles. The polysaccharide nanoparticles were based on HA (MW 1500 kDa), chitosan (MW ≤ 400 kDa, degree of deacetylation 80–95%), and chitosan-*N*-acetyl-l-cysteine (CNAC). The nanoparticles were generated using an electrostatic binding between chitosan or CNAC and TPP or HA/TPP. The size of particles was 17–350 nm and their ζ-potentials varied from −1.4 to +12 mV. The final microparticles (containing nanoparticles) had a slightly positive ζ-potential (2–9 mV) and a microparticle size of 3.0–6.0 µm in diameter. The addition of CNAC increased the ranibizumab entrapment efficiency up to 69% (vs. 29% in the unmodified PLGA microparticles). Presumably, ranibizumab was solubilized by the hydrophilic CNAC, based on thiol group-mediated chemical interactions between *N*-acetyl-l-cysteine and ranibizumab. The use of CNAC improved the release profile of ranibizumab from the PLGA microparticles by minimizing the initial burst release.

Wang et al. [161] formulated ALG-based microspheres with retinoic acid as an active pharmaceutical substance. ALG microspheres 65–115 microns in size were formed by calcium-based cross-linking. The retinoic acid release was stable, slow, and effective for about one month in an in vivo study. The retinoic acid release from ALG microparticles was primarily controlled by microparticle degradation.

Chitosan and HA are the most commonly used polysaccharides for the preparation of nanoparticles for ophthalmic drug delivery. HA is deemed a particularly excellent choice for drug delivery in the posterior segment of the eye. HA-based nanoparticles are mobile in the vitreous gel, and they can reach the retina and choroid [130]. HA has also been used as a coating for other nanoparticle materials. 

Huang et al. [144,163] developed a technology for manufacturing HA-coated (MW 120 kDa) albumin nanoparticles. These nanoparticles were used to deliver Connexin43 mimetic peptide to prevent secondary damage after ischemic and inflammatory diseases of the retina. These nanoparticles had a negative ζ-potential (−44 mV) and small mean size (253 nm) that promoted their diffusion from the vitreous humor to the retina. Coating of the nanoparticles with HA prolonged the drug retention in the target eye area for up to eight weeks. 

Gan et al. [164] designed HA-modified nanoparticles with chitosan and cholesterol. The nanoparticles were prepared by hydrating a dried lipid cholesterol film with a chitosan solution. The nanoparticles were then modified with HA using the binding agents EDC and NHS at pH 4.0 The final HA-modified nanoparticles had mean diameters of 350–450 nm, and their ζ-potential ranged from −20 to −30 mV. The HA-modified nanoparticles were used to study the cellular uptake via interaction with CD 44 receptors. HA with a MW of 200–400 kDa improved the cellular uptake of the nanoparticles, possibly by a CD44-mediated mechanism, whereas HA with a MW of more than 1000 kDa reduced the cellular delivery of nanoparticles.

Andrei et al. [165] synthesized chitosan–gelatin-based nanoparticles for intravitreal cefuroxime delivery using a technique involving double crosslinking in a double emulsion. Nanoparticles of low MW 70 kDa with a deacetylation degree of 91% had a particle size of ≈225 nm and an encapsulation efficiency of about 47%. A peak characterized the profile of cefuroxime release (pH 7.4) in the first hour and a subsequent sustained release followed for 48 h. In vivo studies showed that the cefuroxime released from chitosan–gelatin nanoparticles was eight times more effective than free cefuroxime. 

Glycol chitosan nanoparticles (hydrodynamic diameter 174 nm, ζ-potential 9.6 mV) were used as a matrix for the intravitreal delivery of cerium oxide nanoparticles. Cerium oxide nanoparticles have strong antioxidant and antiangiogenic properties, but their use is limited by their low water solubility [166]. Glycol groups can mask the amino groups of glycol chitosan and block the binding to an anionic vitreous gel [139]. The glycol chitosan nanoparticles significantly increased the aqueous solubility of cerium oxide and, accordingly, its bioavailability, while also reducing cerium oxide toxicity. Intravitreal injections of this type of nanoparticle significantly ameliorated the damage associated with oxidative stress in mouse eyes [166].

#### 4.2.2. Polyplexes

Polysaccharides are also used to bind and deliver polynucleotides, such as RNA and DNA, into cells. Cationic polysaccharides (e.g., chitosan) form nanoparticle-sized complexes (i.e., polyplexes) with anionic polynucleotides. 

Positively charged polyplexes are effectively taken up by the cells, but their poor mobility in the vitreous and ILM limits their cellular delivery [152,153]. However, ocular distribution can be improved with a HA coating. For example, Lee et al. [167] used HA (MW 5000 Da) to coat multimerized siRNA (polysiRNA) polyplexes with branched polyethyleneimine. The polysiRNA polyplex had a size of about 260 nm and a negative ζ-potential of about −5 mV. The polysiRNA polyplex was efficiently distributed through the vitreous and retina and reached the subretinal space [36], since it did not interact with the negatively charged components of the vitreous gel. Presumably, the polyplexes bound to the CD44 receptors on the Müller cells. The polysiRNA polyplexes were non-toxic, and they maintained pharmacological activity after 1 and 7 days.

Martens et al. [168] used HA of various MWs (22, 137, and 2700 kDa) as an electrostatic coating for polyplexes of an anionic pDNA with a cationic *N*,*N*′-cystamine bisacrylamide-4-aminobutanol vector to improve their distribution in the vitreous body. The polyplex charge was rendered negative with a four-fold excess of HA. Polyplexes with low molecular weight HA were mobile in the vitreous (22 and 137 kDa) and provided maximal gene expression (22 kDa).

#### 4.2.3. Self-Assembling Nanoparticles

Micelles can be obtained from amphoteric macromolecules that self-assemble in aqueous solution to form a hydrophobic core and a hydrophilic shell. Water-insoluble drug molecules can be incorporated into the hydrophobic core, while the hydrophilic shell provides stability for the micelles in the aquatic environment [130,138]. 

Koo et al. [151] synthesized self-assembling amphiphilic nanoparticles based on four polymers: polyethyleneimine (MW 25 kDa), glycol chitosan (MW 250 kDa, degree of deacetylation 82.7%), HA (MW 2.34 MDa), and human serum albumin. The hydrophobic component was 5b-cholanic acid. The nanoparticle sizes were approximately the same (210–340 nm), but the nanoparticle ζ-potentials varied from −23 to +33 mV. Strongly positively charged polyethyleneimine nanoparticles interacted with anionic vitreous components and were poorly distributed. Negatively charged human serum albumin and HA nanoparticles demonstrated a broad distribution in the vitreous humor and the retina. Positively charged heterogeneous glycol chitosan and polyethyleneimine/glycol chitosan nanoparticles penetrated through the vitreous humor, but they did not penetrate through the ILM into the retina. The free movement of positive heterogeneous particles in an anionic vitreous gel is possibly related to the anti-fouling effects of glycol groups. This behavior confirmed the correlation between the properties of the nanoparticle surfaces and intravitreal distributions.

### 4.3. Conjugates (Covalent Modification)

The diffusional velocity of molecules in a vitreous gel and their clearance from the eye depends on the molecular size [149]. Thus, the pharmacological effects of intravitreally administered drugs may be prolonged with increasing molecular size. Covalent conjugation to polymers can therefore be used to increase the apparent size of drug molecules [136], making the use of large polymer molecules a rational drug delivery strategy [169,170].

HA has been used to reduce clearance and increase the residence time of drugs in the vitreous humor [136]. Altiok et al. [170,171] conjugated anti-VEGF drug (soluble VEGF receptor; sFlt-1) to HA (MW 300 kDa, 650 kDa, and 1 MDa) to provide sustained release and prolonged retention in the vitreous [170]. The half-lives were about ten times longer for the HA conjugates than for the unconjugated sFlt-1. Conjugation of sFlt-1 with HA did not change the pharmacological activity of this protein drug [170,171]. This study nicely demonstrated the potential use of polymer conjugation in intravitreal drug delivery. Unfortunately, this approach has been very rarely used thus far. 

### 4.4. Intravitreal Implants

Intravitreal implants are based on biodegradable or non-biodegradable polymer containers loaded with active pharmaceutical substances. Intravitreal implants are used for controlled release and prolonged drug action [139,142,157]. Some non-degradable and biodegradable (polylactide- based) implants are already in clinical use, but these products are not based on polysaccharides [172].

Polysaccharides have been utilized experimentally for controlled release in the vitreous. For example, Van Kampen et al. [173] manufactured hollow cylinders (1 mm in diameter, 10 mm in length) of 51 kDa HA that was cross-linked with divinyl sulfone. Diffusional release of proteins from these implants was based on hydrogel swelling and the density of crosslinking. A low degree of swelling and a high crosslink density were achieved by optimizing the ratios of HA and the divinyl sulfone cross-linker. The release of protein from the hollow cylinders was at least 2.5 μg per day for 4.5 months. This system can be used for the delivery of active pharmaceutical ingredients to the retina for the treatment of retinal pathologies.

Some implant designs involve inclusion of drug-loaded nanoparticles in the implant. For example, an ophthalmic implant of zinc-cross-linked HA with bevacizumab-loaded chitosan nanoparticles was fabricated for the treatment of choroidal neovascularization [174]. The bevacizumab-loaded chitosan (50 kDa) nanoparticles were prepared by ionic gelation. The mean size of the nanoparticles was 78 nm, the encapsulation efficiency was 68%, and the loading efficiency was 16%. The in vitro release results showed a prolonged release of bevacizumab from the polymer matrix for two months. However, the role of the nanoparticles is not clear in this context, because bevacizumab acts in the extracellular space, so improved intracellular delivery is not needed. The prolonged release of protein drugs can also be achieved without nanoparticles. 

Manna et al. [155] used chitosan (MW 50–190 kDa and degree of deacetylation > 75%) as a hydrophilic, non-toxic, biodegradable polymer to obtain intravitreal polymer matrices with methotrexate. The chitosan was biodegraded by hydrolytic enzymes to produce amino sugars, which were quickly metabolized. The resulting chitosan matrices were coated with hydrophobic PLA to prolong the release of methotrexate. The implant released methotrexate at the rate of 0.2–2.0 µg/day for more than 50 days.

## 5. Periocular Administration

### 5.1. Subconjunctival Delivery

The subconjunctival space is a widely used route for injections, but regular drug solution injections have a short duration of action. However, the subconjunctival space can also be used for sustained drug release. Drug clearance from the subconjunctival space depends on the molecular size, as small molecules are rapidly lost to the conjunctival systemic circulation, whereas macromolecules are retained longer in the sub-conjunctival space [175]. Particles can be retained for much longer durations in the subconjunctival space than can dissolved molecules; for example, particles larger than 200 nm can be retained for more than 60 days [176].

Ocular drug permeation from the subconjunctival space takes place across the sclera, which is permeable even to polymers. Interestingly, polysaccharides of the same hydrodynamic radius as an antibody have higher permeability coefficients, probably due to their flexible structures. The effective pore size of the sclera has been estimated at approximately 16 nm [177]. After scleral permeation, the compounds reach the ciliary body anteriorly and the choroid posteriorly. For retinal delivery, a periocularly administered drug should also be able to permeate across the RPE, which forms a tight cellular barrier due to tight junctions between the RPE cells [175].

For injectable ocular formulations, slow release of up to several weeks or months is preferable to avoid the need for frequent injections. Crosslinked polymeric systems (inserts, particles) may provide suitable release profiles. A formulation of HA crosslinked with isocyanate-functionalized 1,2-ethylene glycol bis(dilactic acid) was generated and loaded with latanoprost. After subconjunctival administration, latanoprost release was extended for up to 160 days, but complete biodegradation of the material required an additional four months [178].

In-situ gelling systems are a promising class of subconjunctival injectables, as they are liquid at the moment of injection and form a gel in the subconjunctival area, thereby forming a depot for prolonged release of drugs. Several gelling system strategies have been used. For example, Zarembinski et al. [179] developed a hydrogel based on glutathione-modified carboxymethyl HA. Gelation was based on a thiol-disulfide exchange reaction. Gelation occurred within 5 min, resulting in a soft gel that could be used to deliver different cargos, including drugs, proteins, and cells.

Physically crosslinked hydrogels can also be used as formulations for prolonged drug release. Cheng et al. [180] developed a thermoreversible hydrogel based on chitosan, gelatin, and β-glycerophosphate. A latanoprost-loaded hydrogel was formed at body temperature within 1 min. A single subconjunctival injection provided a significant decrease in the intraocular pressure (IOP) within 8 days, and the effect lasted for up to 28 days. In this case, the long delay before the pressure-decreasing effect could prove problematic in a clinical setting. 

Sustained drug delivery is usually used to treat chronic diseases, but it is also valuable as post-surgical therapy. For example, glaucoma filtration surgery requires modulation of the wound-healing process. A carboxymethyl chitosan hydrogel loaded with 5-fluorouracil and bevacizumab, and then in-situ crosslinked with genipin, was injected subconjunctivally to limit scar formation and vascularization of the wound. The hydrogel was nontoxic to the scleral tissue and effectively delayed scar formation [181]. 

Shear-reversible gels do not utilize cross-linking, but they can be injected directly in the gel state because they undergo thinning in response to injection shear. A gel based on carboxymethyl hexanoyl chitosan was capable of forming a dispersion of self-assembled colloids within the gel [182]. Overall, drug release was controlled by the release from the particles and not by diffusion of the dissolved drug in the gel. A single administration of latanoprost-loaded gel lowered the IOP for up to 40 days. 

Methylcellulose and ι-carrageenan dispersions have been evaluated as systems for transscleral delivery of macromolecules [183]. Periocular injection of a gel loaded with antisense oligonucleotides against a junction protein (Cx43) led to a significant increase in the bioavailability in the sclera and choroid when compared with an injection of an oligonucleotide solution. However, in vitro studies did not reveal any enhancement in scleral permeability. Therefore, the positive in vivo result is explained by prolonged drug retention at the site of administration.

Wu et al. [184] studied the tolerability for subconjunctival injections of a mucoadhesive polysaccharide isolated from *Bletilla striata.* This mucoadhesive is a high-MW, nonionic, neutral, and branched polysaccharide that consists of α-mannose and β-glucose monomers. Subconjunctival injection of 10 mg/mL *B. striata* polysaccharide did not cause pathological changes or an inflammatory response, but at higher concentrations (40 or 80 mg/mL), a slight and transient inflammatory response was seen in rabbit eyes. *B. striata* polysaccharide did not cause lesions in the ocular tissues.

Rong et al. [185] prepared an injectable drug delivery system by combining a PLGA-PEG-PLGA hydrogel with insulin-loaded chitosan nanoparticles. Insulin was loaded into the chitosan nanoparticles by ionotropic gelation with TPP. The gel-nanoparticle combination was injected subconjunctivally into rat eyes. The insulin release time was more than 60 days, which was markedly longer than the release periods obtained with the chitosan-based nanoparticles or the PLGA-PEG-PLGA hydrogel alone. Subconjunctival injection of the system did not cause any undesired side effects, including damage to the retinal function, structural changes, cell death in the retina, or glial cell activation. 

### 5.2. Suprachoroidal Delivery

Suprachoroidal injection with microneedles is a relatively new and still experimental mode of periocular drug administration [169]. Suprachoroidal injections are made between the sclera and choroid, thereby avoiding the scleral penetration barrier and sub-conjunctival drug loss to the blood circulation [148]. Retinal bioavailability after suprachoroidal injection is less than that seen after intravitreal injection but higher than after sub-conjunctival delivery [175]. Suprachoroidal injection of a simple drug solution results in rapid drug elimination to the blood circulation, but polymers can be used to prolong the drug retention at the injection site. 

Saline injection results in solution spread over one third of the suprachoroidal space, because this compartment is very thin and limited in space. Addition of a polymer prolongs the retention in the suprachoroidal space and helps to control the solution spread. Rheological properties of the polymer are important in this regard: high MW and moderately non-Newtonian polymeric solutions (e.g., HA) enhance particle spreading, whereas strongly non-Newtonian polymer solutions (e.g., methylcellulose, CMC) are immobilized at the injection site. For example, an injected solution of Discovisc (1.6 MDa HA) is eliminated in one week, while 700 kDa CMC is retained at injection site for 3 weeks [186].

Jung et al. [187] investigated the possibility of targeting formulations to the posterior part of the eye by suprachoroidal space injections of non-mixed particles with HA hydrogel in a single injection. After injection, swelling of the hydrogel pushes the particle suspension to the back of the eye (toward the macula and optic nerve). This approach allowed targeted delivery of 76% of the injected 2-μm particles to the posterior part of the suprachoroidal space. 

## 6. Subretinal Delivery

Subretinal injections are made between the RPE and the neural retina, making this a technically demanding mode of drug administration. Subretinal delivery avoids the barrier of the RPE, as the drug is placed in the immediate vicinity of the neural retina. For example, chitosan microparticles have been used for sustained protein delivery to the suprachoroidal space [172]. Protein was encapsulated into the particles with ionotropic gelation, and the chitosan microparticles showed long-term protein delivery to the retina. However, clustering of the cationic particles induced cytotoxicity at high concentrations [188].

Cationic polymers, such as chitosan and its derivatives, have been widely investigated as gene delivery systems because the polycationic nature of cationic polymers support the condensation of nucleic acids (e.g., DNA, RNA). Sub-retinal injections are the most effective way to administer genetic therapies to the retina. However, limited polyplex diffusion in the vitreous humor and the ILM barrier hamper the efficacy of intravitreal delivery of nucleic acids. 

Mitra et al. [189] used glycol chitosan (MW 250 kDa) for pDNA delivery. Glycol chitosan completely bound pDNA at a 25:1 mass ratio (glycol chitosan/pDNA) and formed ellipsoidal particles with 250 nm hydrodynamic radius and ζ-potential of 25 mV. At 14 days after subretinal injection, the expression of the transgene (green fluorescent protein, in this case) was seen in the RPE cells, while neuronal cells in the retina were not transfected. No retinal toxicity was seen in this experiment.

Oligochitosans (MW 7.3 kDa) were also used for subretinal injections of DNA encoding green fluorescent protein [174]. Although no significant transfection was observed in cultured ARPE-19 cells at N/P ratios of 10–30, transfection of the retinal pigment epithelium and the outer segment of photoreceptors was evident after sub-retinal injection at an N/P ratio of 10. [190]. Oligochitosan (5 kDa) has also been used for siRNA delivery into the RPE [191]. Subretinal injections of polyplexes (N/P 50:1) induced target gene knockdown in a dose-dependent manner in autoimmune uveitis model. 

## 7. Future Aspects 

Polysaccharides clearly have beneficial properties that can facilitate ocular drug delivery, cell therapy, and tissue engineering. Numerous reports have described recent advances, but some concerns and poorly explored aspects remain. For example, biodegradation of polysaccharides in the in vivo situation is difficult to evaluate, and the expression of most degradative enzymes has not been characterized in ocular tissues. Therefore, information about polymer degradation and the rationale for polymer design are not yet optimal. Polymer degradation may influence material removal from the eye [148], and a slower polymer degradation than the rate of drug release may result in empty and unnecessary polymer “ghosts” that will stay in the eye for too long a duration. Therefore, synchronizing the polymer degradation and elimination with the duration of drug release and cell differentiation is of utmost importance. Unfortunately, the design of these systems is hampered by a lack of necessary background information. 

The safety of ocular biomaterials is critically important for the development of drug delivery and tissue engineering materials. Many publications have reported biocompatibility and a lack of toxicity, but these conclusions must be treated with some caution. Typically, toxicity tests involve simple in vitro cell toxicity assays (viability, proliferation, or inflammation), but the situation in the living eye is more complicated. Many subtle mechanisms of toxicity may cause problems during extended treatments. Therefore, detailed and long-term immunological, biochemical, morphological, and functional tests are needed before these materials can be accepted for clinical use. 

## Figures and Tables

**Table 1:** List of FDA-approved polysaccharides used as excipients for ophthalmological drugs [22].

Polysaccharide	Administration ^1^	Drug Dosage Form	Max Potency ^2^	Brand Names
Alginate sodium	T	insert	1 mg	-
Carboxymethyl cellulose sodium	IVT, T	suspension, injection, solution	0.5%	Refresh Tears
Guar gum	T	suspension	0.2%	-
Hyaluronate sodium	IVT	injectable solution	2.3%	Healon, Amvisc, Provisc, AMO Vitrax
Hydroxyethyl cellulose	T	solution, suspension	0.25–1.6%	Rohto Hydra
Hydroxyethyl ethylcellulose	T	solution	0.48%	-
Hyproxypropyl methylcellulose	T	solution, suspension, gel	0.1–0.6%; 2.25% (gel)	Genteal, Nature’s Tears, Tearisol
Methylcellulose	T	solution, suspension	0.05–0.5%	Murocel
Microcrystalline cellulose	IVT	implant	1.66 mg	Retisert
Xanthan gum	T	solution, suspension	0.6%	I-Dew Ultra

^1^ T—topical ophthalmic, IVT—intravitreal; ^2^ maximum potency is the amount of the excipient used in the approved product that is the basis for the IID listing. The potency is given in mg for ocular inserts and in percentage for liquid formulations.

## Abbreviations

ALG	alginic acid or sodium alginate
CMC	carboxymethyl cellulose or sodium carboxymethyl cellulose
CNAC	chitosan-*N*-acetyl-l-cysteine
DED	dry eye disease
HA	hyaluronic acid or sodium hyaluronate
hESCs	human embryonic stem cells
hiPSCs	human induced pluripotent stem cells
HPMC	hydroxypropyl methylcellulose
HUVECs	human umbilical vein endothelial cells
ILM	inner limiting membrane
IOP	intraocular pressure
MW	molecular weight
pDNA	plasmid DNA
PEG	polyethylene glycol
PLGA	poly(lactic-co-glycolic acid)
PNIPAAm	poly(*N*-isopropylacrylamide)
polysiRNA	multimerized small interfering RNA
RGD	adhesion peptide (Arg-Gly-Asp)
RPE	retinal pigment epithelial cells
RPCs	retinal progenitor cells
TPP	sodium tripolyphosphate
TSP	tamarind seed polysaccharides
VEGF	vascular endothelial growth factor
